# *EML4–ALK* fusion transcript is not found in gastrointestinal and breast cancers

**DOI:** 10.1038/sj.bjc.6604341

**Published:** 2008-04-15

**Authors:** Y Fukuyoshi, H Inoue, Y Kita, T Utsunomiya, T Ishida, M Mori

**Affiliations:** 1 Department of Surgery, Medical Institute of Bioregulation, Kyushu University, 4546 Tsurumihara, Beppu 874-0838, Japan; 2 Department of Surgery, Kagoshima University, 8-35-1 Sakuragaoka, Kagoshima 890-8544, Japan; 3 Department of Surgery, Hiroshima Red Cross Hospital, 1-9-6 Senda, Hiroshima 730-8619, Japan

**Keywords:** *EML4–ALK*, fusion transcript, non-small-cell lung cancer, gastrointestinal cancer, breast cancer

## Abstract

Fusion genes have been identified as chromosomal rearrangements in certain cancers, such as leukaemia, lymphoma, and sarcoma. The *EML4–ALK* (*EML4*: echinoderm microtubule-associated-protein-like 4; *ALK*: anaplastic lymphoma kinase) fusion gene has been identified as an oncogene in non-small-cell lung cancer (NSCLC). This study examined the presence of this fusion transcript in gastrointestinal and breast cancers. We evaluated the expression of the *EML4–ALK* transcript in 104 lung cancer cases and in 645 gastrointestinal and breast cancer samples. Only one of the lung cancer samples tested positive for the *EML4–ALK* fusion transcript, whereas none were detected in 555 gastrointestinal and 90 breast cancer cases. Our data suggest that the *EML4–ALK* fusion transcript is not present in gastrointestinal or breast cancers and is specific to NSCLC.

Chromosomal aberrations, in particular, translocations and their corresponding gene fusions, are common occurrences in certain cancers, such as leukaemia, lymphoma, and sarcoma; however, these abnormalities have not been as readily identified in solid cancers (Mitelman *et al*, 2007; Rikova *et al*, 2007). Two characteristics of chromosomal rearrangements, gene fusions, and fusion proteins make them ideal targets for cancer diagnosis and treatment. First, chromosomal rearrangements and gene fusions are specific to tumour cells, are associated with tumour development, and are not found in normal cells and tissues. Target cancer cells are easily detected with high specificity using reverse transcription-polymerase chain reaction (RT–PCR), with primers designed for the corresponding gene fusion partners. Second, treatments targeting fusion proteins are tumour-specific and show fewer adverse effects. For example, imatinib mesylate (Gleevec), which targets the Bcr–Abl fusion protein, is a highly effective treatment for chronic myelogenous leukaemia (Druker *et al*, 1996, 2006). Recently, Soda *et al* (2007) reported the existence of a fusion gene in non-small-cell lung cancer (NSCLC). They reported that a small inversion within chromosome 2p results in the formation of a fusion comprising portions of the echinoderm microtubule-associated-protein-like 4 (*EML4*) gene and the anaplastic lymphoma kinase (*ALK*) gene. This fusion was detected in 5 out of the 75 cases studied. The identification of this and other chromosomal aberrations would be an important step in understanding the mechanisms underlying the development of solid cancers. Therefore, this study was undertaken to evaluate the presence of the *EML4–ALK* transcript in 749 cases of lung cancer and 2 major solid carcinomas (gastrointestinal and breast cancers). We established that the *EML4–ALK* gene alteration is not found in both these carcinomas and is specific to NSCLC.

## MATERIALS AND METHODS

### Clinical samples

Fresh surgical specimens were obtained from 749 patients, as described in Table 1. The patients had undergone surgery at the Kyushu University Hospital at Beppu, Hiroshima Red Cross Hospital, or Kagoshima University Hospital. Written informed consent was obtained from all patients according to the guidelines approved by the Institutional Research Board, and this study was conducted under the supervision of the ethical board of Kyushu University.

### RNA preparation and reverse transcription

Total RNA was isolated using a modified acid guanidinium-phenol-chloroform procedure with DNase. Complementary DNA was synthesized from 2.5 *μ*g of total RNA, as described previously (Inoue *et al*, 1995).

### RT–PCR of *EML4–ALK* fusion transcript

The following primers were designed to amplify 247 bp of the *EML–ALK* fusion transcript (Soda *et al*, 2007): 5′-TGCAGTGTTTAGCATTCTTGGGG-3′ (forward) and 5′-TCTTGCCAGCAAAGCAGTAGTTGG-3′ (reverse).

Either of the fused gene variants (1 or 2) could be amplified with the primer sets described by Soda *et al* (2007).

The primers for nested RT–PCR were also employed: 5′-GCAGTGTTTAGCATTCTTGGGGA-3′ (forward) and 5′-CTTGCCAGCAAAGCAGTAGTTGGG-3′ (reverse).

The forward primer was located in exon 2 of the *EML* gene, whereas the reverse primer was located in exon 3 of *ALK*. Amplification was performed for 35 cycles of 30 s at 95°C, 30 s at 66°C, and 1 min at 72°C. A 10 *μ*l aliquot of each reaction mixture was size-fractionated in 2% agarose gel and visualised by ethidium bromide staining. Amplification of the *GAPDH* (glyceraldehyde-3-phosphate dehydrogenase) gene transcript was used as a positive control (of RNA integrity and assay conditions). *GAPDH* was amplified using the following cycling parameters: 22 cycles of 30 s at 95°C, 30 s at 56°C, and 30 s at 72°C. The following *GAPDH* primer sequences were used: 5′-TTGGTATCGTGGAAGGACTCA-3′ (forward) and 5′-TGTCATCATATTTGGCAGGTT-3′ (reverse) (Inoue *et al*, 1995). The RT–PCR product was cloned into pCR vector using TA Cloning Kit Dual Promoter pCR II (Invitrogen Corp., Carlsbad, CA, USA) and sequenced with ABI PRISM 3100-Avant Genetic Analyzer (Applied Biosystems, Foster, CA, USA).

### DNA extraction

Frozen tissue was dissolved in 200 *μ*g of buffer ATL containing 10% proteinaseK, and genomic DNA was extracted and purified using QIAamp DNA Micro Kit (Qiagen, Hilden, Germany), in accordance with the manufacturer’s protocols.

### Long PCR of *EML4–ALK* fusion gene

The following primers were designed to amplify the genomic sequence flanking the breakpoint of the *EML4–ALK* fusion gene (Soda *et al*, 2007): 5′-CCACACCTGGGAAAGGACCTAAAG-3′ (forward) and 5′-AGCTTGCTCAGCTTGTACTCAGGG-3′ (reverse). Amplification was performed for 35 cycles of 30 s at 94°C, 30 s at 60°C, and 5 min at 72°C. A 20 *μ*l aliquot of each reaction mixture was size-fractionated in 2% agarose gel and visualised by ethidium bromide staining.

## RESULTS AND DISCUSSION

**Figure 1:**
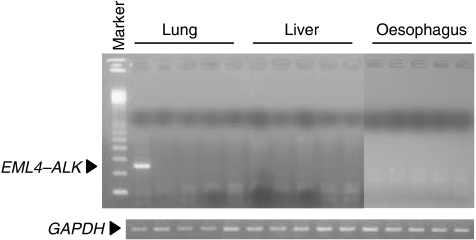
Screening of specimens for *EML4–ALK* mRNA. Representative cases of lung, liver, and oesophageal carcinoma were subjected to reverse transcription-polymerase chain reaction with primers for *EML4–ALK* fusion transcript. Glyceraldehyde-3-phosphate dehydrogenase (*GAPDH*) mRNA is also shown. Marker, 100 bp DNA ladder.

**Figure 2:**
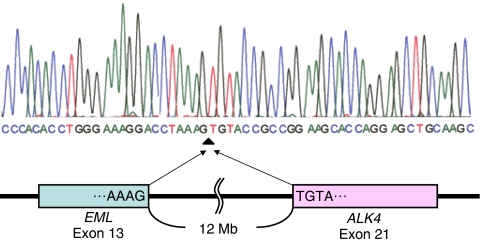
Schematic of reverse transcription-polymerase chain reaction revealing fusion of *EML4* with *ALK* in a case with non-small-cell lung cancer (adenocarcinoma). Line graph shows the position and automated DNA sequencing of the fusion points (identical to the variant 1 transcript).

Expression of the *EML4–ALK* fusion gene was studied in 749 solid tumours (Table 1). A 247 bp transcript was amplified in only 1 out of the 104 lung cancers studied (Figure 1). Sequencing of this transcript confirmed that it was the product of the *EML4–ALK* fusion (type 1 variant) (Figure 2). However, this transcript was not detected in 555 gastrointestinal and 90 breast cancers. The RT–PCR experiments were repeated three times, including one using nested inner primer set; we thereby confirmed the above results. Thus, the *EML4–ALK* fusion gene appears to be specific to NSCLC. Very recently, Inamura *et al* (2008) investigated the presence of *EML4–ALK* fusion in lung cancers: 149 adenocarcinomas, 48 squamous cell carcinomas, 3 large-cell neuroendocrine carcinomas, and 21 small-cell carcinomas. They reported that 5 out of 149 adenocarcinomas showed *EML4–ALK* fusion, but this was not found in carcinomas of other types. The case expressing *EML4–ALK* in our report was also adenocarcinoma. The *EML4–ALK* fusion gene is thus considered to be specific to NSCLC, especially adenocarcinomas.

**Figure 3:**
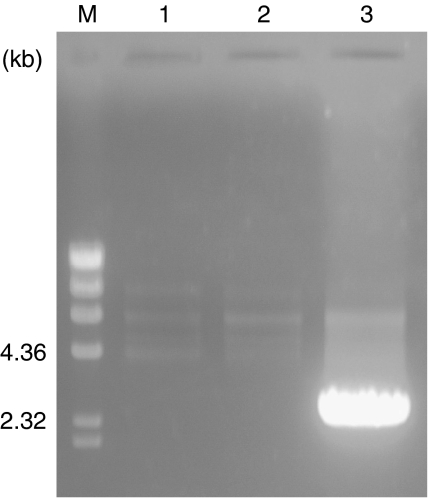
*EML4–ALK* genomic alterations in non-small-cell lung cancer. Results from three representative cases are shown. *EML4–ALK* genomic fusion was found in case 3. Marker, *λ*/*Hin*dIII.

**Figure 4:**
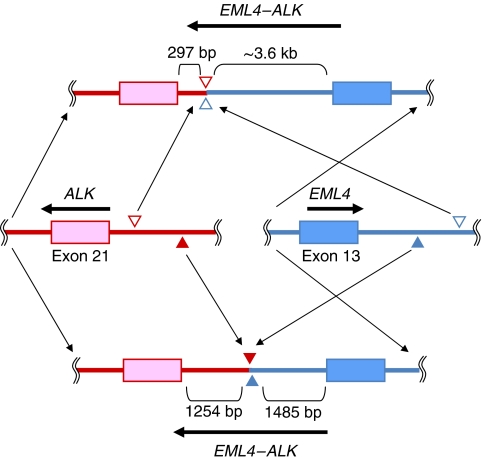
The breakpoints in *EML4* and *ALK* genes. The upper figure shows the structure of *EML4–ALK* variant 1 described by Soda *et al* (2007) and the lower figure shows the structure of *EML4–ALK* that we found. The middle figure shows a wild-type *EML4–ALK*. Filled and open arrows indicate breakpoints.

We next surveyed the genomic alterations in nine cases of NSCLC, including one that was positive for *EML4–ALK* fusion transcript. A 2.5 kb PCR product was detected only in the *EML4–ALK*-positive case (Figure 3). The size of the long PCR product was different from the one originally reported by Soda, indicating that our case had a different breakpoint. The genomic DNA involved by the alteration was sequenced to identify the breakpoints in the *EML4* and *ALK* genes. As a result, *EML4* was disrupted at a position 1485 bp downstream of exon 13 and was ligated to a position 1254 bp upstream of exon 21 of *ALK* (Figure 4). In the report by Soda, *EML4* was disrupted at a position approximately 3.6 bp downstream of exon 13 and was ligated to a position 297 bp upstream of exon 21 of *ALK*. Thus, we confirmed the difference of genomic rearrangements between the present case and the original report by Soda.

Earlier, fusion genes were identified based on the presence of large-scale chromosomal rearrangements. However, genome project completions and advances in microarray technology have revealed chromosomal rearrangements in several kinds of cancers (Mitelman *et al*, 2004; Hirasaki *et al*, 2007). The report of the *TMPRSS2–ERG* fusion in prostate cancer by Tomlins *et al* (2005) is probably the first description of such a gene fusion in solid cancers. This altered gene was recognized in 22 out of 24 examined cases, and activity of the TMPRSS2–ERG fusion protein might be correlated with prostate cancer development. Of course, as this gene does not exist in normal cells, it could prove useful for cancer diagnosis and treatment. The *TMPRSS2–ERG* fusion does not show a chromosomal translocation, but originates from a 2.7 Mb deletion in chromosome 19 (Tomlins *et al*, 2005, 2007). The *EML4–ALK* fusion gene reported by Soda *et al* (2007) is also derived from a genomic inversion of less than 12 Mb in chromosome 2. These alterations (deletions or inversion) are too small to be detected by traditional cytogenetic techniques. Microarrays allow a more detailed analysis and the possibility of detecting novel fusion genes in the absence of chromosomal translocations.

The *TMPRSS2–ERG* fusion gene is specific to prostate cancer, which may be due to the strong induction of *TMPRSS2* by androgen (Perner *et al*, 2006). In contrast, the reason behind the NSCLC specificity of *EML4–ALK* is unclear. This alteration was not detected in two of the most prevalent carcinomas, gastrointestinal and breast cancers. However, as mentioned above, there is a possibility that novel fusion genes will be identified, which can open a new era in the diagnosis and treatment of these common solid cancers.

## Figures and Tables

**Table 1:** Specimens examined for *EML4–ALK* transcript

	**Number**	**Positive case**
Lung	104	1
Colorectum	96	0
Stomach	96	0
Oesophagus	112	0
Breast	90	0
Liver	232	0
Cholangiocellular	11	0
Pancreas	8	0
Total	749	1

ALK=anaplastic lymphoma kinase; *EML4*=echinoderm microtubule-associated-protein-like 4.
